# Facilitators and barriers of managing patients with multiple chronic conditions in the community: a qualitative study

**DOI:** 10.1186/s12889-020-8375-8

**Published:** 2020-02-27

**Authors:** Kah Mun Foo, Meena Sundram, Helena Legido-Quigley

**Affiliations:** 1 Saw Swee Hock School of Public Health, National University of Singapore, 12 Science Drive 2 #10-01, Tahir Foundation Building, Singapore, 117549 Singapore; 2 National University Polyclinics, Singapore, 1 Jurong East Street 21, Singapore, 609606 Singapore; 3 London School of Hygiene and Tropical Medicine, London, WC1H 9SH UK

**Keywords:** Multiple chronic conditions, Qualitative study, Facilitators, Barriers, Primary care physicians, Patients, Caregivers

## Abstract

**Background:**

Approximately one-third of all adults worldwide are diagnosed with multiple chronic conditions (MCCs). The literature has identified several challenges facing providers and patients coping with managing MCCs in the community, yet few studies have considered their viewpoints in combination. A qualitative study involving healthcare providers and users was thus conducted to examine facilitators and barriers of managing patients with MCCs in the community in Singapore.

**Methods:**

This study involves 26 semi-structured interviews with 10 physicians, 2 caregivers and 14 patients seeking treatment in the polyclinics that provide subsidised primary care services. Topic guides were developed with reference to the literature review, Chronic Care Model (CCM) and framework for patient-centred access to healthcare.

**Results:**

Despite the perceived affordability and availability of the support system, some patients still encountered financial difficulties in managing care. These include inadequacy of the nation-wide medical savings scheme to cover outpatient treatment and medications. Half of healthcare users did not know where to seek help. While patients could access comprehensive services in polyclinics, those who did not visit the clinics might not receive timely care. Furthermore, patients reported long consultation waiting time.

Physicians were able to propose and drive quality improvement projects to improve care quality. However, there were challenges to delivering safe and quality care with limited consultation duration due to the need to manage high patient load and waiting time, inadequate communication with specialists to coordinate care, and resource constraints in managing complex patients.

Although providers could equip patients with self-management and lifestyle-related guidelines, patients’ actions are influenced by multiple factors, including work requirements, beliefs and environment.

**Conclusions:**

There were barriers on care access, delivery and self-management. It is crucial to adopt a whole-of-society approach involving individuals, community, institutions and policymakers to improve and support MCC management. This study has also highlighted the importance of considering the different viewpoints of healthcare providers and users in policy formulation and community care planning.

## Background

According to a systematic review conducted in 2011, approximately one-third of all adults worldwide have multiple chronic conditions (MCCs), defined as the presence of more than 1 chronic disease [1, 2]. Various studies across multiple countries, such as Canada, the United Kingdom (UK), the United States (US) and the Netherlands, established the correlation of MCCs with lower self-efficacy, reduced quality of life, and vulnerability to depression and other psychological issues, as well as disability [3–7]. Individuals with MCCs were found to require more medical attention, including a higher number of visits to primary and specialist care. They also had more prescriptions and incurred greater healthcare expenditures compared to those with one or no chronic conditions [1, 8]. In the US, in 2010, US$0.71 out of every US$1 of healthcare expenditures were spent on the management of MCCs [9].

Studies conducted in Canada, the UK, Sweden and Singapore revealed that patients with chronic conditions or MCCs were unable to receive appropriate care in the community. Challenges were encountered in coordinating care, interacting with providers and acquiring adequate relevant information. Some patients had difficulties making decisions and self-managing due to lack of capacity, being more reliant on caregivers, and other barriers at the community, institutional and system levels. Financing expenses related to the management of MCCs were found to be a key problem to be addressed in order to avoid delays in treatment [10–16]. Furthermore, a systematic review found that patients did not always understand their prescribed medication, which could lead to medication error [17]. Despite the benefits of physical activity (PA), Lee (2013) established that US residents with 3 or more chronic conditions were also unlikely to have met PA requirements compared to others with fewer conditions [18].

At the primary care level, studies in the US, Switzerland, Scandinavia, Asia and other regions found problems related to managing chronic conditions. These include fragmentation in the healthcare system, inadequate guidelines, lack of communication among providers, and inability to handle varied and complicated conditions and provide patient-centred care, as well as problems communicating and making decisions on which patients and caregivers agree. General Practitioners (GPs) in the UK, New Zealand, Ireland and Malaysia also reported inadequate consultation time to review and discuss conditions; such consultations are essential to providing quality care [19–22]. Nevertheless, Danielle (2016)‘s study in the US implied that physicians’ satisfaction derived from coordinating care and preventing hospital admission could encourage them to manage MCCs [23].

Through a scoping review, Marie-Eve (2018) identified the provision of patient-centred care, the facilitation of self-management and the training of healthcare staff as common interventions leading to positive outcomes for patients with MCCs [24]. Two other studies in France and Canada illustrated that certain tasks involved in managing chronic conditions could be transferred from physicians to non-physicians within the team, as long as roles were clearly defined [25, 26]. Nonetheless, there is still a lack of effective evidence-based interventions, making it necessary to establish more targeted interventions with greater consideration of patient-centredness in care delivery [27–31].

The literature has pointed to the health system, financing, care management, care coordination and self-management as areas where gaps remain. However, few studies have combined the viewpoints of healthcare providers and users to explore the topic of managing patients with MCCs in the community. With the increasing burden of MCCs and the expectation that chronic diseases will account for 75% of death worldwide by 2020 [1, 32], there is an urgent need to examine aspects of coping with the management of MCCs across various touchpoints, including accessing and receiving care, as well as self-management, in greater detail.

This study aimed to better understand the facilitators and barriers of managing patients with MCCs in the community in a developed country, Singapore. This study specifically covered areas that enable and deter patients from receiving appropriate care from primary care providers and self-managing their chronic conditions. The study incorporated perspectives from healthcare providers, namely, primary care physicians, and healthcare users, including patients and caregivers:

1. From the providers’ perspectives, this study explored the topic of managing patients with MCCs, as well as meeting the varied and potentially complex needs of these patients.
2. Patients’ experiences, as well as caregivers’ experiences with patients accessing community care and coping with their conditions were considered.

The findings, which were triangulated, add value to the literature and should be considered by parties such as policy makers and community care providers to enhance the provision and sustainability of community care.

## Methods

### Study setting

Given its rapidly ageing population, Singapore experienced rising disease prevalence between 2010 and 2017, particularly for hyperlipidaemia, hypertension and diabetes [33]. Older adults aged 60 and above were also found to be impacted by MCCs, with close to 40% of a local study’s respondents informed having 3 or more chronic diseases in 2017 compared to approximately 20% of respondents in 2009 [34].

Singapore’s healthcare financing system is rooted in the value of personal responsibility, coupled with a support system to ensure sustainability and care affordability. This approach enables Singapore’s residents to access care in a timely manner in different healthcare settings without experiencing financial hardship. Individuals can use financial schemes, namely, MediSave, a nation-wide medical savings scheme, to pay for healthcare expenses, as well MediShield Life, a healthcare insurance plan, to fund costly treatments such as hospital stays. Singaporeans who require further financial support can utilise MediFund, a government endowment fund, after exhausting their personal financial resources. There are also other forms of support, such as subsidies for the purchase of medications at restructured hospitals and polyclinics, as well as the Pioneer Generation Package, which was introduced in 2014 to provide further healthcare subsidies to elderly residents who meet eligibility criteria [35–37].

Nevertheless, given the rising demand for healthcare in Singapore, it is essential to continuously review and enhance the care delivery system to provide quality, affordable care and ensure the long-term sustainability of the healthcare industry. The Ministry of Health (MOH) attempted to integrate care by organising healthcare institutions including restructured hospitals, primary care services and other community care services into clusters termed regional health systems. This could facilitate seamless transitions across healthcare settings, for instance, referrals of patients with complex conditions from primary care to restructured hospitals for specialised care, the discharge of patients from hospitals to primary care, and enabling the development of shared-care models between hospitals and primary care. To reduce the utilisation of costly hospital services, the MOH initiated a shift in care focus “beyond hospital to community” [38–40]. This has called for greater community involvement, including that of primary care providers, who are often patients’ first point of contact in the community, to address health-related issues such as chronic disease prevention and management within the community [41].

Within Singapore’s primary care setting, polyclinics that provide subsidised primary care services and made up 20% of primary healthcare have been managing 45% of patients with chronic conditions. On the other hand, private GP clinics, which account for 80% of primary healthcare, have been managing the other 55% of patients with chronic conditions [42]. This ratio shows the imbalance of chronic disease management activities in the community and has raised concerns about the capacity, ability and quality of polyclinics in managing patients with chronic diseases in such a context. This study was thus conducted with physicians who manage patients in polyclinics, as well as caregivers and patients seeking treatment in these facilities, to understand their experience in managing MCCs and to explore the facilitators and barriers of community care.

### Sampling, Recruitment & Data Collection

Twenty-six semi-structured interviews were conducted between October 2018 and February 2019 with 10 polyclinic physicians, 2 caregivers and 14 patients who were managing chronic conditions in 6 polyclinics managed by the National University Polyclinics (NUP) (Table 1).

**Table 1:** **Profiles of participants**

**Physicians**	
	Number of physicians (*n* = 10)
Gender	
Female	6 (60%)
Male	4 (40%)
Years of working in the polyclinic	
< 1–5 years	4 (40%)
6–10 years	1 (10%)
> 10 years	5 (50%)
Care model involved	
Teamlet *(Part of a regular team comprising Family Physicians, Care Manager and Care Coordinator to manage patients with chronic conditions)*	3 (30%)
Non-Teamlet model *(Manages patients of all profiles, both chronic & acute patients)*	5 (50%)
Teamlet & Non-Teamlet model	2 (20%)
**Patients** *(14 interviews were conducted with patients, and 2 interviews were conducted with patients’ caregivers)*	
	Number of patients (*n* = 16)
Gender	
Female	9 (56%)
Male	7 (44%)
Race	
Chinese	15 (94%)
Malay	1 (6%)
Age	
56–60 years old	2 (13%)
61–65 years old	3 (19%)
66–70 years old	8 (50%)
> 70 years old	3 (19%)
Education level	
No formal education	1 (6%)
Primary school	5 (31%)
Secondary school	8 (50%)
Polytechnic	2 (13%)
Employment status	
Retired and/or not looking for job	10 (63%)
Working part time	3 (19%)
Employed with full time job	3 (19%)
Living Situation	
Staying alone	2 (13%)
Staying with family member(s)	14 (88%)
Number of chronic conditions	
2	5 (31%)
3	9 (56%)
> 3	2 (13%)
Years of managing chronic conditions in the polyclinic	
1/2–2 years	4 (25%)
3–4 years	2 (13%)
5 or more years	7 (44%)
Unable to recall the exact duration	3 (19%)

This study undertook purposive sampling by sending email invites to physicians who were involved in managing chronic conditions and who had a minimum qualification of a Graduate Diploma in Family Medicine to participate in the interviews. Upon receiving email replies from physicians, the researcher (FKM) proceeded to schedule for the interview sessions.

Purposive sampling was also adopted to recruit patient and caregiver participants. To be eligible for the study, the patients would need to meet the recruitment criteria of being 40 years old and above, having 2 or more chronic diseases, visiting the polyclinic for 6 or more months and being able to converse in English or Mandarin. In addition, caregivers would have to be involved in the patient’s care management process and are not domestic helpers. The care managers and advanced practice nurses identified patients and caregivers who met the criteria, sought verbal consent and gave the lists of potential participants to the researcher (FKM). The researcher contacted the potential participants through phone calls to arrange for interviews. From the lists given to the researcher, 3 patients were uncontactable, and another 3 refused to participate due to the need to arrange for face-to-face interviews.

Each interview ranged from 30 to 90 min in duration and was conducted in either English or Mandarin. Interviews with physicians were conducted in meeting and consultation rooms, while interviews with patients and caregivers were conducted in patients’ homes, fast food restaurants and cafes. Field notes were documented following the interviews. All interviews were audio-recorded with consent from participants and fully transcribed. Interviews in Mandarin were translated into English. To ensure confidentiality, the participants’ identities were removed and are represented by pseudonyms. All participants were only contacted once for the interviews, and no repeat interviews were conducted. Coding and analysis were conducted after each interview was transcribed. The transcribed data and derived themes were reviewed repeatedly to ensure that all data were taken into account in the themes and sub-themes. By analysing the last few interviews with physicians, as well as with patients and caregivers, it was determined that no new codes were generated. The research team then decided to cease recruitment of participants in February 2019 after reaching thematic saturation and concluding that additional data collection would not derive new codes, themes or relevant information for this study.

### Theoretical framework

The interview topic guides (Additional file 1) were developed with reference to literature review and two frameworks, the Chronic Care Model (CCM) and the framework for patient-centred access to healthcare. The CCM comprises the necessary components to improve care management at the patient, organisation and community levels and has been considered a useful guide to enhance care delivery, leading to improved outcomes. CCM includes 6 elements that affect patient care outcomes, namely, health systems, community, self-management support, delivery system design, decision support and clinical information systems [43]. Furthermore, while care access is vital to health system performance, it would be necessary to consider the supply and demand aspects that could be assessed through 5 dimensions, namely, approachability, acceptability, availability and accommodation, affordability, appropriateness, and the corresponding dimensions of abilities, namely, ability to perceive, ability to seek, ability to reach, ability to pay and ability to engage [44]. The authors jointly developed and reviewed the topic guides to ensure relevance to the primary care context.

In addition, the socio-ecological model (SEM) demonstrated that individuals’ health and practices are influenced by the interplay of individual, interpersonal, community, organisational and policy factors. The SEM is considered relevant to health promotion and disease prevention, and it has been adopted by the Centers for Diseases and Prevention in its initiatives. As various factors contribute to effective chronic disease management in the community, the authors also considered SEM in the process of collecting data and formulating recommendations [45].

### Data analysis

This study adopted an interpretive approach to analysing physicians, patients and caregivers’ responses, and it also considered their experiences. Transcripts were coded using inductive and deductive approaches, and thematic content analysis was conducted with the support of Nvivo12 software. Grounded theory techniques were used, such as line-by-line coding and the identification of emerging and deviant cases. Themes and sub-themes were then derived from the analysis [46].

### Ethical approval

Ethical approval was obtained from the National Healthcare Group Domain Specific Review Board (DSRB), reference number 2018/00825. Prior to starting the interviews, all participants were briefed on the study objectives and details as stated on the information sheet and signed consent forms for participation in the study. All data collected have been stored securely.

## Results

**Fig. 1:**
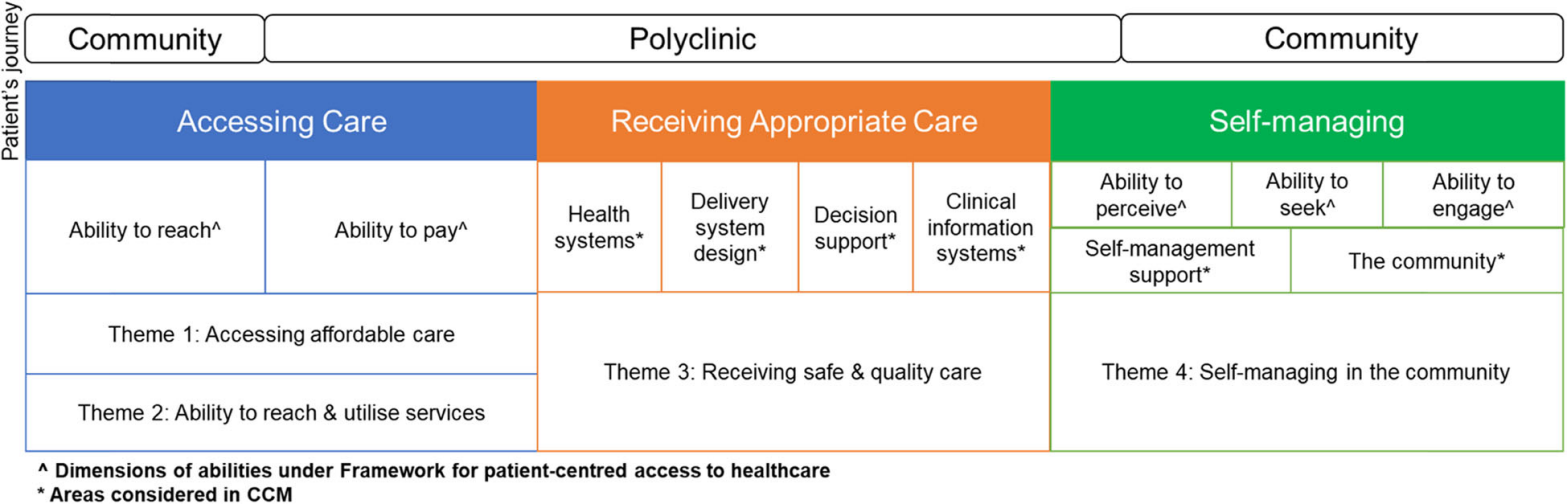
Journey of patients with MCCs & derived themes

Taking reference from the CCM and framework for patient-centred access to healthcare and considering the interview findings, the patients’ journey in managing MCCs generally involves 3 key areas, namely, accessing care, receiving appropriate care and self-managing [43, 44] (Fig. 1). First, patients need to access healthcare and other essential services without experiencing financial hardship, and able to physically access the services that they need. Second, it is crucial for patients to receive appropriate care at the polyclinic. Last, patients have to be able to self-manage with minimal monitoring by healthcare professionals in the community.

Findings through interviews with healthcare providers and users were merged under each theme, with a clear distinction between the facilitators and barriers. The 4 themes, specifically accessing affordable care, the ability to reach and utilise services, receiving safe and quality care, and self-managing in the community, as well as the corresponding 17 sub-themes indicated below each theme, were elaborated as follows.

### Theme 1: accessing affordable care

- Facilitators

i. Affordable charges & presence of “helping hands”

Physicians indicated that the most common chronic conditions that are managed in polyclinics include diabetes, hypertension and hyperlipidemia. All patients in this study reported being diagnosed with at least one of these conditions. A total of 10 patients mentioned that they chose to manage chronic conditions in the polyclinics due to subsidised and affordable charges. Other reasons quoted for visiting polyclinics include close proximity to their homes and relationships with healthcare providers. Most patients tapped into MediSave to pay for the charges [47]. Providers shared that patients requiring financial and other support could be referred to financial counsellors or medical social workers situated in polyclinics to facilitate the process of subsidies and other applications.

b. Barriers

i. Financial challenges specific to the MediSave withdrawal limit, higher non-standard drug charges & support schemes

Several patients reported the inadequacy of the MediSave scheme to cover outpatient visits, particularly with higher charges for certain medications. Physicians shared that although unsubsidised non-standard medications might be beneficial for patients with chronic diseases, they would not be able to prescribe them to patients with financial constraints. For existing patients who were prescribed non-standard medications but subsequently encountered financial challenges, physicians expressed that they would reduce the dosage or replace non-standard medications with other drugs, with consideration of the clinical implications. A patient commented that he would follow his neighbour to purchase medication from a neighbouring country if needed to cope with high medication costs.

Although patients could apply for financial assistance through polyclinics, Dr. Candy emphasised that some might not be eligible:

***“They (patients) don’t meet the criteria to get the subsidy but yet…they really feel that they can’t cope (with) the charges, but there is nothing (that) you can do because the criteria have already been set.”***

Dr. Amanda also questioned the necessity for all needy patients to be assessed based on eligibility criteria:

***“I don't see why people who need a walking stick must prove that they need it. Is there a need to prove that they are already 80 years old?... Must we fill up some forms for them to qualify for (purchase of) wheelchair?”***

Dr. Amanda further suggested for flexibility to be exercised on a case-by-case basis and added that healthcare providers would rather pay for the patients’ expenses when needed.

Half of the caregiver and patient participants reported not knowing where they could seek help, with a few sharing that they would discuss their situations with their family members. While one of the patients, Mary, applied for a Foreign Domestic Worker grant about one month ago to hire a helper to take care of her elderly mother with chronic conditions, she stressed on the need to reduce processing turnaround time to ensure the provision of timely support:

***“It shouldn't take so long. By the time you (the government) approved the grant, my mother might no longer be around…I think the service can be a little faster.”***

### Theme 2: ability to reach and utilise services

- Facilitators

i. Perceived accessibility to comprehensive services

Physicians reported that patients could access consultation, diagnostic and pharmacy services to manage MCCs in a single polyclinic. As services such as physiotherapy and podiatry are not available at certain polyclinics due to space and resource constraints, some patients may need to travel to a different polyclinic to access these services. However, all patients reported that with the availability of buses and trains, they did not encounter any challenges traveling to polyclinics. Physicians explained that patients’ frequency of visits to polyclinics depends on their ability to control their diseases instead of the number of conditions and could range from 2 to 4 times a year. Patients can schedule subsequent appointments during each visit in the polyclinics and receive SMS reminders about these appointments.

b. Barriers

i. Impractical for polyclinics to cover all patients

Some participants reported challenges facing bedridden patients and wheelchair users in trying to access polyclinic services.

Physicians further cautioned that patients might “fall through the cracks” if they did not schedule any follow-up appointments or defaulted on their appointments, for instance, due to work commitments. Dr. Peter explained:

***“Most of them will say it is (due to) work…they forget…still have their medications... if they are not here, I can't help them anyway.”***

While polyclinics can follow up with patients enrolled under specific care teams or programmes, physicians generally felt that it would be challenging to reach out to all other patients.

ii. Challenges of elderly navigating the polyclinic system

A few participants highlighted the difficulties encountered by some elderly in their consultation journeys. These include the challenges of them using self-registration and payment kiosks, communicating with polyclinic staff who do not speak dialects, and missing stations. Ang, who is an elderly patient, mentioned the following:

***“It is very troublesome now, we (elderly) don’t know how to read and use the kiosks…If my daughter did not go with me, I will not know how to press (the kiosks). In the past, when we buy medicine, we pay by cash, now we (will) need to place cash in the machine.”***

iii. Long waiting time

Half of the patient and caregiver participants reported long consultation waiting time of up to 3 h despite having scheduled appointments. Several patients thus stressed waiting time in the polyclinics as a key area for improvement. Two patients mentioned that the demand for polyclinic services had increased with new housing developments around the polyclinics and because of patients who continue to seek treatment at the polyclinics even after relocating to other areas. For instance, a patient shared that due to personal preference and familiarity, he has continued to visit Queenstown Polyclinic, which is located in the West, despite moving to the East.

### Theme 3: receiving safe and quality care

- Facilitators

i. Quality assurance and improvement efforts

Physicians reported that in order to have the skillsets to deliver safe and quality care, they are required to attend regular Continuing Medical Education and training sessions conducted either by specialists or physician champions. Furthermore, a physician mentioned that by tracking common clinical indicators across polyclinics, physicians could identify potential gaps and initiate quality improvement (QI) projects. A few physicians were of the opinion that the polyclinics had created a facilitating environment for physicians to propose and drive QI projects.

ii. Collaboration among multidisciplinary teams

Close to half of the patients interviewed were visiting nurses instead of physicians regularly to review their conditions and were generally supportive of this approach. Mary said that:

***“Yes, it is a nurse instead of the doctor who does the explanation. The nurse is really good…She has more time to explain the details to us.”***

Physicians could also refer patients with multiple medications to clinical pharmacists to assist with medication reconciliation. A few physicians illustrated the crucial roles played by non-doctors in understanding and addressing the needs of patients, including those who require more assistance in making changes.

Through the interviews, physicians described multiple care delivery models in the polyclinics, of which the teamlet model was the most discussed. Physicians explained that there are plans to expand the teamlet model and empanel more patients through this model to comprehensively address healthcare needs. Five physicians interviewed were involved in this model, which comprises a team of 2 doctors, care manager and care coordinator. While doctors review patients’ laboratory results and assess conditions, trained nurses, who assume the roles of care managers, conduct counselling sessions to educate and empower patients to control and manage their conditions. The counselling sessions could cover education on chronic diseases, training on insulin injection for diabetes patients, taking and tracking of blood pressure and blood sugar readings, as well as guidelines on lifestyle practices. In addition, care coordinators who are lay-persons support by keeping track of the screening tests that patients are due for, as well as assisting in appointment scheduling and tracking. With fixed care teams managing specific patient groups, physicians elaborated that provider-patient relationships could be well established. In addition, Dr. Christine shared the following:

***“If there are any difficult patients, we can always discuss within the team on how to manage.”***

b. Barriers

i. Challenges of physicians adhering to clinical and other guidelines

Although physicians could refer to clinical practice and other guidelines to understand the latest care standard and targets, a physician mentioned that it would be difficult to refer to such guidelines, particularly during consultation sessions. She felt that it would lower patients’ confidence if physicians paused to check guidelines during the consultation process. Another physician also raised the need for the timely updating of guidelines to ensure alignment between national and international guidelines to avoid confusion.

ii. Perceived inadequate consultation duration

Most physicians reported the issue of high patient load as a key barrier to providing appropriate levels of care to their patients. Physicians also needed to manage patient waiting time, which has been tracked as an operational key performance indicator (KPI). Physicians mentioned that they could only spend an average of 10 min with each patient diagnosed with chronic conditions. Many felt that this amount of time was inadequate, particularly for patients with MCCs who were referred from hospitals. To manage patients discharged from hospitals, physicians explained that they needed time to review the discharge summary and access different IT systems to view clinical notes and lists of medications.

Physicians shared that the duration of consultations for some patients might be reduced due to several reasons. These include the presence of patients perceived as “highly demanding” or “overly empowered,” with a long list of questions and requests. There were also instances of IT system break-down and slowness, further inducing time pressure and stress among physicians. It was reported that inadequate consultation durations might create the possibility for error and result in physicians neglecting critical areas, which would be detrimental to patient care. As Dr. Amanda said:

***“You cannot rush a chronic patient's consult (session). If they (the polyclinics) just pile patients to the queue…you are bound to make mistakes.”***

iii. Lack of care continuity

Some patients visit both hospitals and polyclinics concurrently, but physicians reported several challenges in providing coordinated care. First, while physicians can view the clinical notes of patients visiting or referred from hospitals using the same electronic medical record (EMR) system, they are unable to view the detailed notes of other patients. Second, it was reported that most primary care physicians (PCPs) and specialists mainly communicated through hardcopy memos passed through patients’ hands. PCPs highlighted the challenges of contacting specialists involved in co-managing patients in a timely manner, with less than half mentioning that they could liaise with specialists through emails or phone calls. With the presence of these constraints, it could be challenging for polyclinics to coordinate care for patients who use healthcare services across primary and hospital settings, as Dr. Jenny recalled:

***“My patient was double-dosing himself with the medication stocked by the specialist, but we continued to give it because we didn't know that patient was seeing a specialist and his medicine was changed.”***

iv. Difficulties of polyclinics managing complex patients

Moreover, physicians reported various challenges of managing certain patient groups, including hospital-referred patients. Frail elderly with MCCs, individuals with poorly controlled conditions, bed-bound patients, and others with rare diseases or complex conditions such as end-stage kidney failure were flagged. Physicians specifically highlighted the lack of expertise, infrastructure and resources, as well as the unavailability of specific medications and services such as occupational therapy for post-stroke patients. For instance, Dr. John explained:

***“The main challenging part would probably be the medications because we are not trained to give some medications… and the ministry will have to provide us with the resources… to see the patient safely.”***

Physicians reported that some patients may choose not to manage their conditions in hospitals due to certain considerations, such as cost, the challenge of traveling to the hospital, and disagreement with hospital care plans. Although it may not be optimal for such patients to visit polyclinics, physicians reported that they would continue to manage them and discuss their cases with multidisciplinary teams or consult specialists when needed. Physicians would also refer patients in deteriorating condition to hospitals.

### Theme 4: self-managing in the community

- Facilitators

i. Patient education and empowerment

Some physicians explained that they would request for more information from new patients in order to better address those patients’ potential challenges in coping with chronic disease management. These details, which include medical history, family background, daily routines and risk factors, would help physicians assist patients in setting targets such as exercise hours. All patients and caregivers reported that healthcare professionals had provided them with dietary and exercise guidelines and that they were able to understand the information.

Some patients reported that healthcare providers had guided them to self-monitor their blood pressure and blood sugar levels and capture the readings regularly on a form to be discussed with providers at upcoming consultation sessions. A few patients mentioned that the providers had also informed them about symptoms to take note of and that they had been advised to seek early treatment when those readings were out of the standard range.

ii. Patients with understanding on conditions and making some forms of lifestyle modifications

Most healthcare users were able to articulate patients’ conditions. Patients reported that when unwell, they would visit their polyclinics and GP clinics to seek treatment. To obtain further information on their conditions, the majority mentioned that they would either check with healthcare professionals or discuss with their family members. Most patients emphasised making some form of lifestyle change, mainly through reducing food and sugar intake and engaging in physical activities. Six patients reported participating in community programmes such as running, cycling and yoga.

b. Barriers

i. Infeasible for polyclinics to track patients’ progress closely

Physicians reported that they were checking laboratory results and clinical indicators to infer whether patients were making any lifestyle changes and would refer patients to other providers, such as nurses and dieticians, to reinforce the guidelines when needed. However, Dr. Peter explained that due to resource limitations, polyclinics were not able to customise detailed plans for individual patients or monitor the progress closely. He mentioned the following problem:

***“There is no service to assess what kind of exercise (patients) are suitable for. Nobody to prescribe the exact exercises (that) they need, nobody to monitor their progress.”***

ii. Low adoption of technology by patients

Although patients and caregivers could check their screening test results prior to consultation sessions through HealthHub [48], an online application, only one person reported doing so. Key reasons for not using the application include the details being available only in English and in small font size, not being able to interpret the results, as well as preference for healthcare providers to explain the results to avoid anxiety.

While polyclinics offer telecare services, through which patients could measure and submit their blood pressure and blood glucose readings online for nurses to monitor and provide necessary advice, participants reported that this might only benefit patients with IT knowledge. For instance, Leong held the view that:

***“I think the government spends a lot of money on technological services. I think that is good. But the problem is, some people (who) know how to use it will benefit from it. But those who don’t will be at a disadvantage.”***

iii. Multiple factors influencing patients’ self-management and decisions to make lifestyle changes

Although patients could take greater ownership of their health by self-monitoring their conditions, a physician noted that some might not be able to afford devices such as blood pressure monitor and blood glucose monitor, and consumables. Physicians and healthcare users emphasised that patients would also need to be able to interpret the readings and recall the standard guidelines, including dietary control. In addition, most patients mentioned that they had at some point forgotten to take their medication and would simply continue with the next dose of medication. A few physicians also reported that patients’ work nature was a key contributor to missing medications.

Patients’ lifestyle behaviour were reportedly affected by various factors. First, five healthcare users mentioned that patients and family members would search for information online. However, one physician cautioned that the information might be unreliable, and she had tried to clarify the details with patients. Second, the environment around patients’ homes and workplaces, as well as the nature of their work, could affect their food choices and decisions to make lifestyle changes. For example, dietary choices could be dependent on the availability of affordable healthy food options near homes and workplaces. In addition, Ah Hock, a taxi driver, felt that his work nature was a key reason for not being able to exercise regularly.

***“Because we (drivers) have to cover our rental and petrol before talking about earning, so sometimes struggle for certain hours… when I come back, I’m tired already.”***

Third, even though patients could be aware of the benefits of physical activities, they might not be able to exercise due to physical constraints, as explained by Patrick:

***“The only thing that affects me is that my leg hurts…Exercising is good, but it may affect my leg. I don’t know who to ask about it? Not sure what’s the problem.”***

Lastly, patients’ and caregivers’ beliefs, for example, in terms of perceived benefits and adverse outcomes of making lifestyle changes, might influence their decision to do so. Linda, a caregiver to her mother-in-law who was above 70 years old, said:

***“She (patient) started smoking since she was young. There’s a saying that, old people if they suddenly stop smoking, they will go faster.”***

## Discussion

### Key Findings & Recommendations

This study has explored facilitators and barriers to care access, delivery and self-management, taking into account healthcare users’ and providers’ experiences. In terms of care access, the majority of participants said that the provision of affordable care in the polyclinics was a key facilitator but still reported financial barriers that could deter patients from receiving timely support in the community. Patients also reported long waiting time in the polyclinics, and some might not seek treatment regularly. To deliver safe and quality care, the polyclinics created a facilitating environment to encourage physicians to embark on quality improvement initiatives, and created multi-disciplinary teams with established roles and responsibilities. However, it is still crucial to address challenges related to resource constraints and the lack of capability to manage MCCs, which could deter providers from providing an appropriate level of care to patients. In addition, this study found providers attempting to facilitate self-management by empowering patients and increasing their involvement in self-care. Nevertheless, patients had difficulty adhering to the guidelines, as their practices are generally influenced by multiple factors.

Table 2 summarises the themes and sub-themes derived from this study. It is essential to consider the facilitators and address key barriers to scale up chronic disease management activities in the community.

**Table 2:** Themes and sub-themes

Themes	Sub-themes	
	Facilitators	Barriers
Accessing affordable care	• Affordable charges & presence of “helping hands”	• Financial challenges specific to the MediSave withdrawal limit, higher non-standard drug charges & support schemes
Ability to reach & utilise services	• Perceived accessibility to comprehensive services	• Impractical for polyclinics to cover all patients • Challenges of elderly navigating the polyclinic system • Long waiting time
Receiving safe and quality care	• Quality assurance and improvement efforts • Collaboration among multidisciplinary teams	• Challenges of physicians adhering to clinical and other guidelines • Perceived inadequate consultation duration • Lack of care continuity • Difficulties of polyclinics managing complex patients
Self-managing in the community	• Patient education and empowerment • Patients with understanding on conditions and making some forms of lifestyle modification	• Infeasible for polyclinics to track patients’ progress closely • Low adoption of technology by patients • Multiple factors influencing patients’ self-management and decision to make lifestyle changes

First, a previous study in Singapore involving hypertensive patients found that some patients were not supported adequately despite the availability of various financing schemes [16]. This led to late treatment or other financial hardship as a result of seeking treatment. Another study in Singapore conducted with healthcare providers suggested a reform to healthcare financing to provide affordable and appropriate care for patients with complex conditions [49]. In addition to these studies, our study, which incorporated the view of both healthcare providers and users, specifically explored the issues of affordability in the primary care setting and raised questions about the need to revamp the financing framework, particularly to meet the needs of patients with MCCs and visits to multiple healthcare providers. Based on studies of individuals with MCCs in other countries, those with more chronic conditions spend more on healthcare due to a higher number of healthcare visits [1, 8]. In Singapore’s context, although the MOH set a standard MediSave withdrawal limit of up to $500 [50], patients with more complex and poorly managed conditions could incur higher healthcare charges. While there are support schemes to facilitate patients in accessing timely care without exposure to financial hardship, such as the Medication Assistance Fund to support needy patients who require non-standard drugs [51], our study questions the adequacy of these schemes in supporting patients who do not meet the eligibility criteria. It may also be useful for the government to work with agencies and community partners to communicate key schemes and application processes to healthcare providers, patients and caregivers and continue to review the eligibility criteria. Based on this study, approximately half of the healthcare users were not aware of where they could seek help.

Second, studies identified the lack of care accessibility and other access issues, such as the absence of 24-h services, as a key barrier in primary care [42, 52]. Another study established that patients with chronic diseases, especially those with more than one chronic condition, had a higher likelihood of receiving delayed treatment [53]. This study revealed that even though most patients could travel to polyclinics using public transport and therefore access comprehensive services at the polyclinics, a few patient groups might still be unable to receive timely care management. These include patients with mobility limitations, those who do not schedule follow-up appointments, and those who miss their appointments. While it may be challenging for polyclinics to reach out to patients with chronic conditions who do not seek treatment, it may be useful for polyclinics to extend collaborations with other healthcare and social care providers to follow up with such patients.

Third, through interviews and surveys with healthcare providers, several studies in various countries have identified the issues of inadequate capacity and consultation time in the primary care setting [19–22, 42]. Our study further affirmed these challenges, which might affect patient safety and care quality, based on interviews with physicians, patients and caregivers. Physicians highlighted inadequate consultation duration as an existing barrier to managing patients with MCCs and those referred from hospitals with multiple medications. Both healthcare users and providers further shared the issue of rising demand for polyclinic services, which might potentially reduce future consultation duration. Moreover, patients and caregivers highlighted the need to improve waiting time for consultations, which is also tracked as one of the physicians’ KPIs. These discussions emphasised the importance of reviewing and matching the demand and supply of polyclinic services. To address the capacity issues in polyclinics and allow polyclinic physicians to spend more time managing patients with conditions of higher complexity, the government might consider further increasing the involvement of non-physicians, for instance, through the polyclinics’ teamlet model. To date, the Singapore government has also established primary care networks to provide necessary support to encourage more private GPs which made up of 80% of primary healthcare, to manage patients with chronic conditions [42, 54]. Furthermore, the government has introduced the Community Health Assist Scheme, which entitles residents from middle- and lower-income groups to subsidised care at GP clinics [55]. The government could consider assessing the effectiveness of such initiatives in supporting private GPs to embark on chronic disease management and determine any additional support needed.

A potential way to increase primary care capacity would be through the adoption of technology to manage chronic conditions and lifestyle activities [56]. However, 2 studies found a lower technology adoption rate among older adults [57, 58]. Likewise, this study found low adoption of technology to check screening results among healthcare users interviewed. Additionally, participants reported that elderly face challenges when using technology to navigate the polyclinic systems. Although healthcare institutions are increasingly tapping onto technology in care delivery and monitoring processes, it may still be essential to continue “human intervention” in managing and meeting the needs of elderly patients, as a qualitative study’s participants in the Netherlands found that technology should support instead of replacing care delivery [57]. We would also propose for polyclinics and other providers to potentially reach out to caregivers who are more IT savvy to support patients in tracking their online health records and interpreting the results.

Through this study, several physicians emphasised the lack of expertise, resources and infrastructure to manage complex patients, such as frail elderly with MCCs and end-stage diseases. While the general direction should be to manage patients safely in the community and reduce the use of costly hospital services in Singapore, it is crucial to consider the types of patients who are actually manageable in the polyclinic or community context and whether resources are adequate to provide appropriate care. For instance, physicians would need to be trained and given sufficient time to assess the needs of complex patients and equipped to prescribe certain uncommon medications.

Two studies involving elderly patients indicated the lack of communication and coordination among healthcare providers, leading to “threats” to patient safety and “hassles” in the care management process [11, 12]. In terms of communication between PCPs and specialists, this study found that less than half of the PCPs could liaise directly with specialists who refer or are co-managing patients. PCPs were also not able to view full medical records of patients seen at hospitals that use a different EMR system. These findings imply the need to establish a direct communication platform between primary care and hospitals, particularly to discuss referral cases. To minimise the number of referral cases that are too complex to be managed in polyclinics, forums involving specialists and PCPs could be conducted to foster understanding of polyclinic patient profiles, care delivery processes and limitations. Patients and caregivers could also be more involved by informing their respective healthcare providers about visits to other providers or new prescriptions.

In addition, patients in the community generally spend most of their time self-monitoring and making lifestyle choices. Thus, patients should be equipped with the right skillsets for self-care. Various studies identified the need to facilitate self-management and provide adequate support, for example, through community programmes [59–66]. Although healthcare providers might have provided self-management guidelines and support to polyclinic patients, our study found that patients’ practices were still affected by their daily activities involving work and the community, as well as their beliefs and physical condition. It is thus suggested for government institutions to increase their efforts of involving communities and workplaces to co-create a healthy living environment, which includes making healthier food options available [67, 68]. Community providers and peers may potentially be involved in influencing the choices of patients participating in community programmes and assisting them in monitoring conditions. Moreover, the government could engage employers to provide adequate support to employees with chronic conditions.

In contrast, our study revealed a potential scenario whereby patients could be very involved in their care, make the effort to search for additional information and discuss this with the providers. While it would be beneficial for patients to be more involved in these discussions, consultation sessions might be lengthened. This highlights the need for government and polyclinics to continue reviewing patient loads and the appointment time slots allocated to each patient. Healthcare providers might also attempt to direct patients and caregivers to credible online resources to ensure accurate understanding of conditions.

**Fig. 2:**
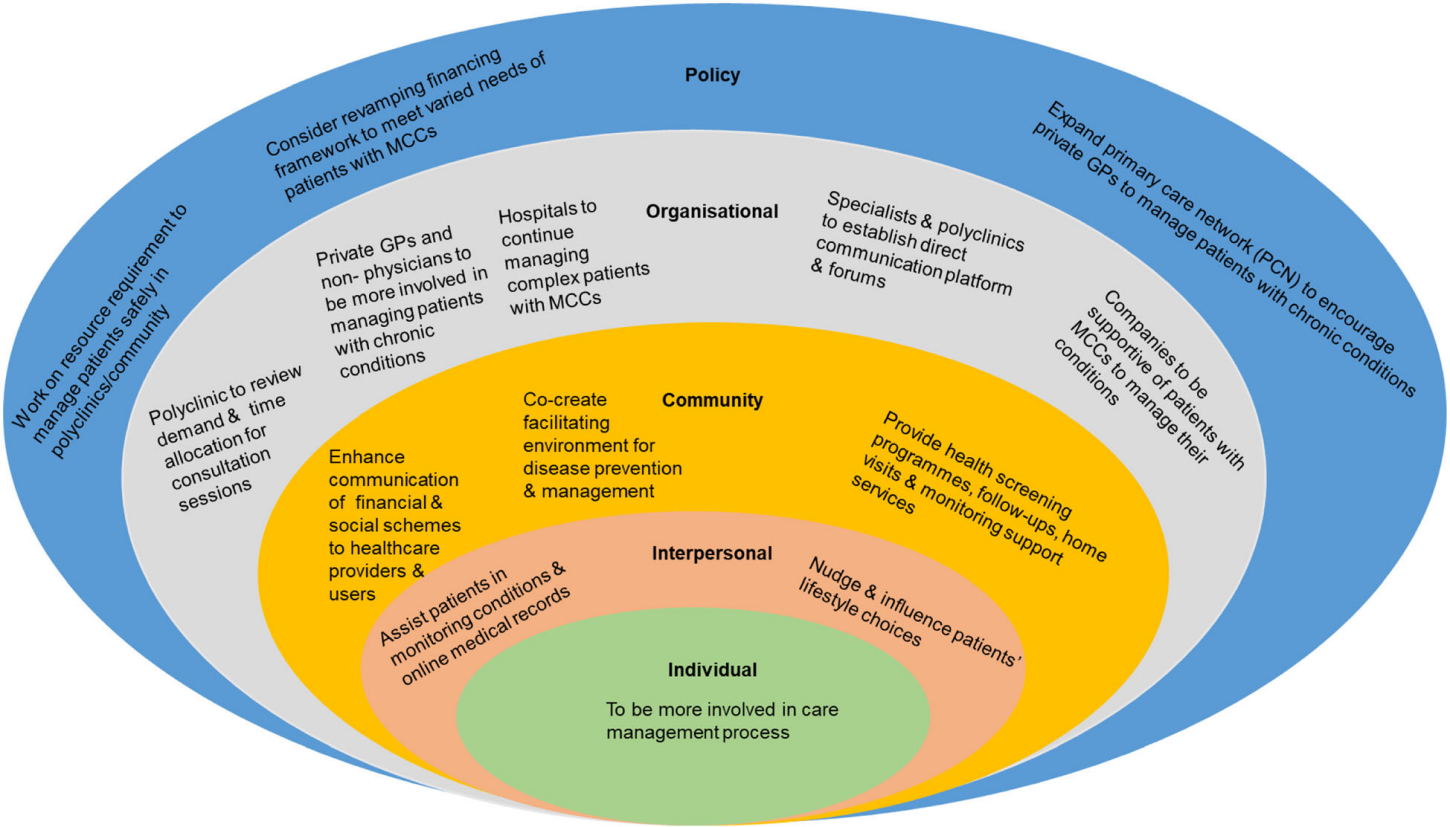
Socio-ecological model depicting the whole-of-society approach to manage MCCs

Through this study, the elements of CCM and the framework for patient-centred access to healthcare were found to have addressed patients’ touchpoints in accessing and receiving care, and self-managing comprehensively. In coherence with these models, our findings also demonstrated the need to involve multiple stakeholders to extend efforts to address gaps and scale up positive aspects of respective components such as health systems, delivery systems, self-management, community support and care access. Specifically, this would involve a whole-of-society approach in accordance to socio-ecological model to examine several areas, as shown in Fig. 2 [45].

### Strengths and limitations

To-date, no other qualitative study in Singapore has explored the topic of managing polyclinic patients with MCCs in the community. In this study, we interviewed both care providers and users with different perspectives to understand the barriers and facilitators of managing MCCs. The interview topic guides were developed with reference from the established model and framework, namely, CCM and the framework for patient-centred access to healthcare. This ensured that questions pertaining to care access, delivery and self-management were addressed extensively. Some participants had also shared information about their experience beyond the interview questions. Furthermore, this study managed to capture the viewpoints of both genders, the elderly and those of lower socioeconomic status. Half of the physicians interviewed had been working in the polyclinics for 10 or more years.

Although there are also patients managing chronic diseases with private GPs’ support, this study was only conducted in the polyclinic context. In addition, while there are other providers, such as nurses and dieticians, involved in managing chronic diseases, this study only interviewed physicians. Limitations might also exist due to biases. First, selection bias could be a problem, as patient and caregiver participants were mainly identified and referred by care managers and advanced practice nurses. Second, social desirability bias might be present, with most healthcare users mentioning that they had made some forms of lifestyle change following the detection of diseases and that they would like to be more involved in managing their healthcare needs when these topics were explored. The study could not recruit more working adults below 55 years old, nor did it include patients from other ethnic groups, with only one Malay patient being interviewed. While we were not able to interview more of these patients, physicians generally shared their encounters.

Given these limitations, this study could be scaled up both locally and internationally to incorporate the viewpoints of other stakeholders, including other healthcare providers, such as private GPs, nurses, allied health professionals, policy makers and working adults with MCCs. A new study may also be initiated to explore the delivery of sustainable, safe and quality care, especially for healthcare institutions facing resource constraints. Moreover, it could be beneficial to look into funding models to meet the varied needs of patients with different health and social status.

## Conclusion

It is crucial to look into sustainable approaches for care delivery to address rising healthcare demand and the growing burden of chronic diseases. While the Singapore government has planned to shift the focus of care beyond hospital to the community, PCPs highlighted the challenges of managing rising numbers of patients with chronic conditions, providing safe and quality care due to limited capacity in polyclinics and inadequate communication between specialists and PCPs. Furthermore, even though polyclinics attempted to empower patients to take greater ownership in managing their conditions, patients’ ability to adopt the recommended practices is influenced by various factors, such as community and work nature. These findings emphasised the need to take on a whole-of-society approach that looks beyond collaboration with healthcare-related stakeholders for management of MCCs, as well as to consider varying standpoints of various stakeholders and potential implications to further contribute to policy formulation and community care planning.

## Supplementary information

**Additional file 1.** Interview Topic Guides. Interview Topic Guides for (1) Patients and Caregivers, and (2) Physicians.

## Data Availability

The datasets generated and/or analysed during the current study are not publicly available due to consideration of data sensitivity. We also did not obtain written consent from the study participants and ethical review board to share the datasets. As such, we are responsible for ensuring confidentiality of the collected data and are unable to disclose any additional data.

## Abbreviations

CCM: Chronic Care Model
KPI: Key Performance Indicator
MCCs: Multiple Chronic Conditions
NUP: National University Polyclinics
PCPs: Primary Care Physicians
SEM: Socio-ecological Model
