# Supplementary material for: Facilitators and barriers of managing patients with multiple chronic conditions in the community: a qualitative study
Source: BMC Public Health. 2020 Feb 27;20:273. doi: 10.1186/s12889-020-8375-8 (PMC7045577; doi:10.1186/s12889-020-8375-8)
Supplement: Supplementary file 1 — Additional file 1. Interview Topic Guides. Interview Topic Guides for (1) Patients and Caregivers, and (2) Physicians. [file 12889_2020_8375_MOESM1_ESM.docx]

**Interview Topic Guides**

1. **Topic Guide for Patients & Caregivers**

*[Questions asked were dependent on the conversation with interviewees]*

| **No.** | **Topic** | **Question** |
| --- | --- | --- |
| 1 | Patient’s background | 1. To request & capture patients’ demographic details:  - Age, gender, ethnic group, highest education level, occupation, residential area, housing type, living arrangement  1. [For caregiver] Could you share your roles as a caregiver to your ward? 2. Can you share about your/your ward’s existing medical conditions? 3. What condition(s) are you/your ward seeking treatment in the polyclinic? 4. How long have you/your ward been visiting the polyclinic to manage your/your ward’s condition(s)? 5. How did you/your ward start managing your/your ward’s chronic condition(s) in the polyclinic? 6. How did you get to know that you/your ward could manage chronic condition in the polyclinic? 7. Why did you/your ward choose to manage your/your ward’s condition(s) in the polyclinic? 8. Do you/your ward seek treatment elsewhere for other condition(s) or to manage the same condition(s)?  - Why? - Please provide details on the institution(s), frequency of visit and condition(s) being managed at the respective institution(s).  1. Do you/your ward seek non- conventional treatment, e.g. Traditional Chinese Medicine (TCM)?  - Why? - Please elaborate on the institution(s), frequency of visit and condition(s) managed, supplements taken (if any) |
| 2 | Care management process in the polyclinic | 1. What did the polyclinic check with you/your ward during your first visit?  - Do you remember who you/your ward had spoken to?  1. What did the polyclinic tell you/your ward during your first visit?  - Did the polyclinic explain your/your ward condition to you/your ward? - Did the polyclinic provide you/your ward with details on available treatment options or services?  1. Did the polyclinic doctors/staff explain to you/your ward on how your/your ward’s condition will be managed in the polyclinic?  - Did you/your ward understand the details? - Did you/your ward agree with the plan? - Were there any changes to the plan subsequently?  1. Could you explain about your/your ward’s typical journey of a visit to the polyclinic?  - Who did you/your ward meet in each touch point? - Did you/your ward see the same healthcare staff during your return visits? - What did the healthcare staff ask/tell you/your ward? - What did you like/not like about your/your ward’s journey in the polyclinic?  1. How did the polyclinic involve you/your ward in the care management process? 2. How would you/your ward like to be involved in the care management process? 3. How many times did you/your ward visit the polyclinic in a year? 4. What was the duration of each visit? 5. How long did you/your ward usually wait for the following services?  - Lab test - Consultation - Collection of medication - Others  1. Generally, what was the total duration of your/your ward’s consultation session for chronic condition? 2. How is your/ward’s relationship with the healthcare staff in the polyclinic?  - Are there any healthcare staff that are in charge of managing your/your ward’s care and understand you/your ward well?  1. Do you think the polyclinic was able to understand your/your ward’s healthcare needs?  - Why?  1. Was the polyclinic able to meet your/your ward’s healthcare needs?  - Why?  1. What do you think about the quality of healthcare services in the polyclinic? |
| 3 | Facilitating self-management | 1. Did the polyclinic provide any plan/guidelines/information to you/your ward on how to manage your ward’s condition (e.g. medication, symptoms etc.) when in the community?  - What information was provided? - How was the information being communicated to you/your ward? - Were you/your ward able to understand the information? - Did you/your ward find the information useful? - Were you/your ward able to follow through the information? - How did you/your ward apply the information in your/your ward’s daily life?  1. Did the polyclinic encourage and help you/your ward to set care goals, e.g. in terms of exercising, diet, quitting smoking (if applicable)?  - Please provide more details of how the care goals were developed. - Did anyone in the polyclinic explain to you/your ward on how you/your ward could monitor the meeting of care goals? - Did the polyclinic staff go through the care goals with you/your ward regularly?   - How? - Do you think you/your ward have/will be able to meet your/your ward’s goal?   - Why?  1. Did the polyclinic inform you/your ward of ways that you/your ward can contact the healthcare providers if you/your ward encountered any issues? |
| 4 | Self-management in the community & care coordination | 1. How have you/your ward been involved in managing your overall healthcare needs? 2. Do you have a caregiver?  - Who is your caregiver? - What are the roles of your caregiver?  1. Are you a caregiver to someone? [This question will be answered by caregiver participants at the start of interview]  - Who are you taking care of? - What are your roles as a caregiver?  1. How were you/your ward reminded to attend your/your ward’s appointment?  - Have you/your ward ever missed your appointment? Why?  1. How many types of medication do you/your ward need to take each time? 2. How were you/your ward reminded to take your/your ward’s medication?  - Have you/your ward ever missed your/your ward’s medication? Why?  1. Do you/your ward take any non-conventional medications, e.g. those prescribed by TCM?  - Please provide more details.  1. Did you/your ward make any lifestyle changes since detection of the condition(s)?  - If yes, how and in what way? - How did you/your ward obtain the necessary information to facilitate the process in making lifestyle changes? - Otherwise, why not?  1. If you/your ward have multiple appointments in polyclinic and hospital/other institution, how do you/your ward:  - Manage your/your ward’s appointments? - Keep track of medications to be taken?  1. If applicable, could you share about the challenges that you/your ward encountered in seeking care from different healthcare providers? 2. How are you/your ward coping with your condition in the community?  - How much do you know about your/your ward’s conditions? - Do you/your ward know what symptoms to look up for? - Where do you/your ward obtain information to cope with your/your ward’s condition? |
| 5 | Health-related support system in the community | 1. Did any polyclinic staff contact you/your ward before/ after your consultation in the polyclinic?  - If yes, who had contacted you/your ward? - How did the staff contact you/your ward, e.g. through phone call, home visit? - What did the staff mention to you/your ward during the conversation?  1. Who have you/your ward approached when you/your ward have any questions about your health conditions?  - During office hour - After office hour  1. Did the polyclinic refer you/your ward to any programmes or services in the community?  - Please elaborate.  1. Have you/your ward participated in any other health related programmes in the community, e.g. exercises?  - What programme(s) have you/your ward participated in? - How did you/your ward know about the programme(s)? |
| 6 | Adoption of technology | 1. How did you/your ward book your/your ward’s appointment for follow up in the polyclinic?  - Have you tried booking your/your ward’s appointment online? - If yes, through what platform(s)?  1. Did you/your ward check your/your ward’s medical record online?  - If yes, through what platform(s)? - Were you/your ward able to understand the information? - Do you think viewing of online medical record help you/your ward in terms of managing your/your ward’s condition?   - How?  1. Could you share your/your ward’s experience of using the online application(s) for appointment booking and checking of medical record?  - If needed, in what way do you think the online systems can be enhanced?  1. What are your thoughts towards using technology in your/your ward’s care management process, e.g.  - For appointment booking - Checking of medical record |
| 7 | Affordability & accessibility to healthcare services in the polyclinic | 1. Affordability    - How did you/your ward pay for consultation fee and fees for other services in the polyclinic, e.g. cash, Medisave?  - Have you/your ward encountered any challenges in paying for medical charges in the polyclinic?   - In what way?   - Have you/your ward been refused care previously due to financial issues?   - If needed, who did/would you/your ward approach when you/your ward require additional support or services, e.g. welfare services or grant application? - Do you think you/your ward have received adequate support?   - Were you/your ward affected financially due to the need to pay for your/your ward’s healthcare services? - If yes, how? - Are you/your ward holding onto CHAS/PG cards? - If yes, how are you/your ward using these cards? - What do you/your ward think about these cards? - Do you think there are adequate support, in terms of financial and social services in the community?  1. Accessibility  - How do you/your ward travel to polyclinic for your/your ward’s appointment? - Are you/your ward encountering any difficulties of traveling to polyclinic for your/your ward’s appointment? Please elaborate. |
| 8 | Success factors & other challenges | - 1. Did you/your ward encounter any other challenges that deter you/your ward from seeking regular care from the polyclinic & other healthcare providers?   2. Are you satisfied with the care that you/your ward have received in the polyclinic? - What aspects have worked well? - What aspects would need to be improved? - Would you recommend anyone to seek treatment for chronic conditions in the polyclinic?   1. Overall, do you think you/your ward are coping well with your conditions while in the community? - Why?   1. What do you think can be done by the following parties to improve healthcare services in the community? - Polyclinics - Other healthcare provider(s) - Government - Patients - Caregivers - Other parties   1. Are there any other matters that you would like to highlight? |

**Annex D: Topic Guide for Physicians**

*[Questions asked were dependent on the conversation with interviewees]*

| **No.** | **Topic** | **Question** |
| --- | --- | --- |
| 1 | Physician’s background | 1. Could you share briefly about your background & healthcare experience, e.g.  - Previous work experience - No. of years with the polyclinic - Years involved in managing chronic patients - Roles and responsibilities in the polyclinic  1. Why did you choose to work in a polyclinic? 2. Are there any specific groups of patients, e.g. those with certain conditions that you are managing? 3. What are the typical profile(s) of patients that you are managing? |
| 2 | Availability of polyclinic services | 1. What is the polyclinic’s operating hour? 2. What are the existing chronic conditions that are being managed in polyclinic?  - What are the more common conditions?  1. Could you share on the services available in this polyclinic to manage chronic patients?  - Are there any services that chronic patients require but not available in the clinic? |
| 3 | Care model to manage chronic patients in the polyclinic | 1. What is the estimated proportion of patients with more than one chronic condition in your polyclinic? 2. How do you identify chronic patients that come for regular follow up after the first visit?  - Do you tag patients with chronic conditions in the electronic medical record system for identification purpose?  1. Could you elaborate on the care model to manage chronic patients?  - How did you develop the care model? - Are there any differences in care models to manage patient with one chronic condition and those with more than 1 condition? - Are there any other initiatives & programmes to manage chronic patients in your polyclinic?  1. Do chronic patients always see back the same physician/care team in their return visits?  - Why?  1. Did you review the care model to manage chronic patients?  - How often did you conduct the review? - What factors did you consider in the review process? - Did you make use of any data in the review process? - If yes, what type of data?  1. What do you think about “right-siting’ effort, e.g. patients referred from hospital to primary care?  - Please elaborate on the care model for this group of patients. |
| 4 | Care management process in the polyclinic | 1. What are the information that you would request from patients during their first visit?  - How are these information being used in patient care process?  1. What is the process to assess and understand patients’ needs?  - What are the areas that are being considered? - Do you have and need to adhere to any guidelines?   - Do you think these guidelines are useful?   - What are the enablers and challenges of adhering to the guidelines?  1. Do you develop patient care plans?  - If yes, how do you develop the care plans? - How are patients involved in the process of developing the care plans?   - What details (if any) do you provide patients in the process to facilitate their decision-making process? - How do you communicate patient care plan to them?   - How do you ensure that patients understand the care plan?   - What actions would you take if patients are not agreeable to the plan? - Do you track the progress of the care plans? - How?  1. Do you have a care team in the polyclinic to manage chronic patients?  - If yes, what are their roles? - Do you think these individuals can take on bigger roles?   - In what way?  1. About how many patients do you see in a day?  - How many are chronic patients? - How many are acute patients?  1. What is the duration for 1 consultation session?  - Chronic patient? - Acute patient?  1. Could you explain the typical journey of a chronic patient’s visit to the polyclinic?  - Would you know roughly how long patient need to wait for the following?   - Lab test   - Consultation   - Collection of medication   - Others - Generally, how long does a chronic patient spend in the polyclinic in 1 visit?  1. How often do chronic patients need to return to polyclinic for follow up? 2. How do the chronic patients book their appointments in the clinic? 3. How important do you think it is to establish relationship with patients?  - How do you do so? |
| 5 | Facilitating self-management | 1. Do you provide any plan/guidelines/information to facilitate patients (both proactive and passive patients) to self-manage and monitor their conditions in the community?  - If yes, what are the information provided? - Who communicate the information to patients? - Were there any patients that encountered challenges in:   - Understanding the information?   - Adhering to the plan?  1. Do you encourage and help patients (both proactive and passive patients) to set care goals?  - How do you do so? - How do you monitor the progress of patients achieving the goals?  1. Do you actively encourage patients (both proactive and passive patients) to use online platforms?  - If yes, for what purpose? - Did your patients share their experience with regards to usage of the online platforms?  1. Did your patients (both proactive and passive patients) encounter any challenges to self-manage and monitor their conditions in the community?  - Please elaborate on the challenges, e.g. adherence to medication - What can be done to overcome the challenges? |
| 6 | Support system in the community | 1. Are there any existing collaborations between the polyclinic and the community with regards to programmes or services?  - Have you referred chronic patients to any of these programmes or services?  1. Are there any programmes or services required by chronic patients but you are unable to refer patients to or not available in the community currently? 2. Do you follow up with patients before/after their consultation in the clinic, if yes,  - For which group of patients? - Through what platform? - For what purpose? |
| 7 | Care Coordination & escalation process | 1. How do you work with your colleagues to coordinate care for patients within the polyclinic? 2. Is there any process in place to manage chronic patients visiting both polyclinic and other healthcare providers for multiple conditions? 3. Are you able to view electronic medical record of patients seeking care elsewhere?  - What do you think about the system? - If needed, what are the plans to enhance the system?  1. How often do you communicate with specialists?  - If yes, for what purpose? - What are the communication platforms?  1. Do you also communicate with other healthcare providers?  - Who do you communicate with? - How often? - For what purpose?  1. What do you think have worked well for care coordination? 2. What are some challenges encountered in coordinating care?  - How can this be improved?  1. What is the process to manage patients with deteriorated conditions? |
| 8 | Affordability & Accessibility to healthcare services | 1. Have your patients encountered any challenges in terms of the following? Please provide some examples.  - Paying for medical charges in the polyclinic? - Visiting the polyclinic for regular follow up? - Other challenges?  1. How do you ensure that patients would not encounter financial risk due to cost incurred to seek treatment for medical condition? 2. Do you think all patients be entitled to same care access?  - Why?  1. How do you ensure that there is equity in providing care to patients to ensure that provision of healthcare services is based on needs instead of affordability? 2. Do you think there are adequate support system, in terms of financial and social services in the community, particularly to meet the needs of patients with chronic conditions? |
| 9 | Organisation’s plan & direction to provide quality care | 1. How do you work with your other colleagues in the polyclinic? 2. What are the platforms for discussion and information exchange? 3. Could you share some of NUP/the polyclinic’s plan to ensure and promote safe and high-quality care, e.g. in terms of:  - Facilitating reporting and handling of errors? - Motivating clinicians to provide quality care? - Facilitating care coordination within and across organisations? - Others?  1. Could you elaborate on how NUP/the polyclinic trains the various groups of staff to provide quality care to patients with chronic conditions?  - Physicians - Nurses - Administrators etc.  1. How do you monitor the performance of the care team? |
| 10 | Success factors & other challenges | 1. What aspects of managing patients with chronic diseases have worked well? 2. What aspects have not worked well? 3. Do you think chronic patients are receiving adequate support to manage their conditions in the community? 4. Did you receive adequate support in the process of managing chronic patients?  - Did you encounter any challenges in the process?  1. Are there any patient groups that are highly challenging to be managed in polyclinic?  - Please elaborate on the:   - Typical profiles of patients   - Challenges faced   - Plans to overcome the challenges  1. What do you think can be done by the following parties to address the challenges?  - Patients - Caregivers - Community - Organisation, i.e. NUP - Government |
